# Lactose Induces Phenotypic and Functional Changes of Neutrophils and Macrophages to Alleviate Acute Pancreatitis in Mice

**DOI:** 10.3389/fimmu.2018.00751

**Published:** 2018-04-17

**Authors:** Li-Long Pan, Yuan-Yuan Deng, Ruxing Wang, Chengfei Wu, Jiahong Li, Wenying Niu, Qin Yang, Madhav Bhatia, Gudmundur H. Gudmundsson, Birgitta Agerberth, Julien Diana, Jia Sun

**Affiliations:** ^1^School of Medicine, Jiangnan University, Wuxi, China; ^2^State Key Laboratory of Food Science and Technology, Jiangnan University, Wuxi, China; ^3^Nutrition and Immunology Laboratory, School of Food Science and Technology, Jiangnan University, Wuxi, China; ^4^Wuxi People’s Hospital Affiliated to Nanjing Medical University, Wuxi, China; ^5^Inflammation Research Group, Department of Pathology, University of Otago, Christchurch, New Zealand; ^6^Biomedical Center, University of Iceland, Reykjavik, Iceland; ^7^Division of Clinical Microbiology, Department of Laboratory Medicine, Karolinska Institutet, Karolinska University Hospital Huddinge, Stockholm, Sweden; ^8^Institut National de la Santé et de la Recherche Médicale (INSERM), Unité 1151, Institute Necker-Enfants Malades (INEM), Centre National de la Recherche Scienctifique, Unité 8253, Paris, France; ^9^Université Paris Descartes, Sorbonne Paris Cité, Paris, France

**Keywords:** lactose, inflammation, immunoregulation, neutrophils, macrophages

## Abstract

Acute pancreatitis (AP) is one common clinical acute abdominal disease, for which specific pharmacological or nutritional therapies remain elusive. Lactose, a macronutrient and an inducer of host innate immune responses, possesses immune modulatory functions. The current study aimed to investigate potential modulatory effects of lactose and the interplay between the nutrient and pancreatic immunity during experimentally induced AP in mice. We found that either prophylactic or therapeutic treatment of lactose time-dependently reduced the severity of AP, as evidenced by reduced pancreatic edema, serum amylase levels, and pancreatic myeloperoxidase activities, as well as by histological examination of pancreatic damage. Overall, lactose promoted a regulatory cytokine milieu in the pancreas and reduced infiltration of inflammatory neutrophils and macrophages. On acinar cells, lactose was able to suppress caerulein-induced inflammatory signaling pathways and to suppress chemoattractant tumor necrosis factor (TNF)-α and monocyte chemotactic protein-1 production. Additionally, lactose acted on pancreas-infiltrated macrophages, increasing interleukin-10 and decreasing tumor necrosis factor alpha production. Notably, lactose treatment reversed AP-associated infiltration of activated neutrophils. Last, the effect of lactose on neutrophil infiltration was mimicked by a galectin-3 antagonist, suggesting a potential endogenous target of lactose. Together, the current study demonstrates an immune regulatory effect of lactose to alleviate AP and suggests its potential as a convenient, value-added therapeutic macronutrient to control AP, and lower the risk of its systemic complications.

## Introduction

Acute pancreatitis (AP) is a common clinical abdominal disease with an increasing incidence worldwide and has become the leading cause of hospital admission for gastrointestinal disorders in many countries (1). AP begins with intra-acinar trypsinogen activation and autodigestion of the pancreas, which led to local inflammation (2). When the local inflammation becomes uncontrolled, it may lead to systemic inflammatory responses with impact on multiple organs including the lungs which are ultimately responsible for AP-associated mortality (3, 4). Despite extensive efforts to clarify its etiology and pathophysiological mechanisms, specific pharmacological and nutritional therapies of AP remain elusive. Therefore, strategic therapeutic or nutraceutical interventions to limit local pancreatic inflammation, prevent, or treat diverse AP-associated complications are highly needed.

Both conventional views and recent studies have highlighted the importance of acinar cell inflammation, innate immune cells, and derived mediators in the pathophysiology of AP (5–7). Following acinar cell damage and release of inflammatory cytokines/chemokines, neutrophils, and macrophages, two deleterious immune cell types in the early stage of AP, infiltrate the pancreas, which are early hallmarks of the disease (8). Neutrophils, macrophages, as well as dendritic cells with distinct cell surface and intracellular markers have been associated with the development and severity of acute inflammatory conditions (9–12). A balance between pro-inflammatory and anti-inflammatory mediators produced from acini and immune infiltrates is critical for the modulation of AP. Tumor necrosis factor (TNF)-α, monocyte chemotactic protein (MCP)-1, and interleukin (IL)-10, known as cytokines/chemokines are important in the development of AP (10, 13). Severity of pancreatitis is associated with increased pancreatic content of Ly6C^hi^ monocytes/macrophages and is dependent upon the expression of TNF-α by these cells (10). MCP-1, a member of the C-C chemokine family, exerts strong chemoattractant activities in monocytes, macrophages, and lymphocytes. Treatment targeting MCP-1 has been shown to exert protective effects against AP (13, 14). Consequently, modulation of immune cell activation and inflammatory cytokine/chemokine production may be a rational approach to alleviate AP.

Lactose is a β-galactoside consisting of galactose and glucose residues, the main carbohydrate in mammalian breast milk. Lactose has demonstrated modulatory effects on regulatory T cells, THP-1 monocytes, and macrophages *in vitro* (15, 16) and its treatment has been evaluated in immunological conditions, including necrotic enteritis, *Plasmodium berghei*-induced pulmonary infection, and sepsis with mixed implications (17–19). The anti- or pro- inflammatory effects of lactose seem to be related to its concentrations and primary molecular targets in the context of disease. Galectin-3 is a α-galactoside-binding lectin distributed in epithelial linings of many organs and in various inflammatory cells especially macrophages (20, 21). Galectin-3 chemoattracts monocytes/macrophages and marks macrophage activation (20, 21). The interaction of lactose with particular galectin members largely determines the types of immune responses elicited during inflammation.

In this study, we aimed to address the question of whether lactose exerts modulatory effects on innate immune responses, in particular those mediated by innate immune cells and cytokines, as well as on acinar cell responses during experimental AP. In addition, the potential molecular target of lactose has also been determined.

## Materials and Methods

### Animals

Female BALB/c mice of 6- to 8-week-old (Su Pu Si Biotechnology Co., Ltd., Suzhou, Jiangsu, China) were maintained in specific pathogen-free environment at the Animal Housing Unit of Jiangnan University (Wuxi, Jiangsu, China) under a controlled temperature (23–25°C) and a 12-h light/dark cycle. All animals were provided with standard laboratory chow and water *ad libitum*. A period of 1 week was allowed for animals to acclimatize before any experimental operation was undertaken. All animal-related experimental protocols were approved by the Institutional Animal Ethics Committee of Jiangnan University in compliance with the recommendations of national and international guidelines for the Care and Use of Laboratory Animals, and were performed in accordance with the guidelines therein. Five to eight animals were used in each group for the experiments and have been specifically indicated in the respective figure legends.

### Induction of AP

Mice were randomly assigned into control or experimental groups with eight animals in each group. Animals were given 3, 6, and 10 hourly intraperitoneal (i.p.) injections of normal saline or saline containing caerulein (50 µg/kg, Sigma, St. Louis, MO, USA) as previously described (8). In our regimen, lactose (Sigma, St. Louis, MO, USA) at a dose of 100 mg/kg was given i.p. to mice either half an hour before or 1 h after the first caerulein injection as prophylactic or therapeutic experimental groups, respectively. 1 h after the last injection, animals was sacrificed with a lethal dose of pentabarbitone sodium (20 mg/mL). Harvested blood was centrifuged (3,000 *g*, 10 min, 4°C), serum was collected, and stored at −80°C. Freshly harvested samples of pancreas were weighed, dried in oven (80°C, 48 h), and reweighed to determine pancreatic edema. Tissue samples including pancreas and lungs were stored at −80°C for subsequent experiments.

### Serum Amylase Measurements

Serum was collected by allowing the blood to coagulate at ambient temperate for 25 min, and subsequently centrifuged at 3,000 *g* for 10 min at 4°C. The supernatant was then collected for analysis. An iodine-starch colorimetric method was used to measure serum amylase levels as previously described (22). Briefly, serum samples were incubated with a prewarmed substrate buffer (Jiancheng Bioengineering Institute, Nanjing, Jiangsu, China) for 7.5 min at 37°C. Absorbance was measured, following addition of 0.5 mL iodine and 3 mL ddH_2_O to the mixture, at 660 nm using a UV-2450 UV–VIS spectrophotometer (Shimadzu Corporation, Kyoto, Japan).

### Myeloperoxidase (MPO) Activity

Myeloperoxidase activity was evaluated using an MPO assay kit (Jiancheng Bioengineering Institute, Nanjing, Jiangsu, China) according to the manufacturer’s protocol as previously described (22). Pancreatic tissues in 0.9% saline or homogenization medium provided by the MPO assay kit (1:19 mass/volume) were homogenized by a homogenizer (IKA, Staufen, Germany). Five percent of pancreatic tissue homogenates (200 µL) and chromogenic reagent (3 mL) were added to a sterile tube, and after mixing, it was put in water bath for 30 min at 37°C. Stop solution (provided by the assay kit) was added to the above mixtures to stop the reaction. Absorbance was then analyzed at 460 nm within 10 min and MPO activities are expressed as units/g tissue.

### Histology Examination

Histological examination was performed as previously described (8). Ten hours after AP induction, freshly harvested pancreatic and lung tissues were fixed with 4% paraformaldehyde overnight. The tissues were then dehydrated with gradient ethanol solutions and embedded in paraffin blocks and cut into 5 µm sections. The sections were dewaxed in xylene, hydrated through upgraded ethanol solutions, and subsequently stained with hematoxylin and eosin (H&E). Pancreatic and lung injuries were examined under a DM2000 light microscope (Leica Microsystems GmbH, Wetzlar, Germany) at 200× magnification.

### ELISA

Tissue homogenates of mice receiving different treatments as well as isolated primary cells, including acinar cells, peripheral neutrophils, and peritoneal macrophages were assayed for inflammatory mediators using specific sandwich ELISA kits (Biolegend, San Diego, CA, USA). Previous procedures validated by our group were adopted, according to the manufacturer’s protocols. Absorbance was measured at 450 nm within 30 min, using an automated microplate reader (Multiskan™ GO; Thermo Fisher Scientific Oy, Vantaa, Finland). Concentrations of various inflammatory cytokines or chemokines were calculated based on the absorbance measurements, and are expressed as pg/mL.

### Flow Cytometry

Female BALB/c mice were treated with caerulein together with or without lactose and 3 h later cells from the pancreas were recovered. Single cell suspensions were stained for 30 min at 4°C after FccRII/III blocking in phosphate-buffered saline (PBS) containing 2% fetal calf serum, 0.5% EDTA, and 0.1% sodium azide. Surface staining was performed with the following monoclonal antibodies (mAbs): anti-CD45 (eBioscience, clone 30-F11), -F4/80 (eBioscience, clone BM8), -CD11b (eBioscience, clone M1/70), -CD11c (eBioscience, clone N418), -IL-10 (eBioscience, clone JES5-16E3), -TNF-α (eBioscience, clone MP6-XT22), -Ly6G (eBioscience, clone 1A8), -CD62L (eBioscience, clone MEL-14), -TCRβ (eBioscience, clone H57-597), and -CD19 (eBioscience, clone 1D3). For determining macrophages and neutrophils, cells were surface stained with anti- F4/80, -CD11b, -Ly6G, -CD62L, and for B and T cells, cells were stained with anti-CD45, -CD11b, -TCRβ, and -CD19 followed by incubation for 30 min. For cytokine expression by macrophages, cells were stained with the following mAbs: anti-TNF-α, -IL-10. Stained cells were analyzed on a Becton Dickinson Fortessa flow cytometer.

### Isolation of Peripheral Blood Neutrophils

Female BALB/c mice were treated with caerulein together with or without lactose, and 3 h later blood samples of mice were drawn using anticoagulant tubes and diluted with 2 mL PBS. Neutrophils were purified using density gradient centrifugation through Ficoll-Paque Plus. Contaminating erythrocytes were removed by red blood cell lysis buffer. Cells were resuspended in RPMI 1640 medium and allowed to recover at 37°C in a humidified 5% CO_2_ incubator and incubated with lipopolysaccharides (LPS, 1 µg/ml, Sigma-Aldrich, St. Louis, MO, USA) for 3 h before ELISA assays.

### Isolation of Primary Peritoneal Macrophages

Primary peritoneal macrophages were isolated as previously described (23) with modifications. Female BALB/c mice were treated with caerulein together with or without lactose and 3 h later mice were anesthetized with 10 mg/mL of pentabarbitone sodium and closed peritoneal lavage was performed using 10 mL ice-cold PBS. Peritoneal exudate cells were collected, washed with PBS, and resuspended in supplemented Dulbecco’s Modified Eagle Medium (DMEM). Peritoneal macrophages were allowed to adhere in 6-well plates at 37°C in a humidified 5% CO_2_ incubator, incubated with LPS (1 µg/ml) for 3 h, and then subjected to subsequent ELISA assays.

### Primary Acinar Cell Preparation

Pancreatic acinar cells were freshly prepared from mouse pancreas by collagenase treatment (24). In brief, pancreas from three mice were removed, infused with digestive buffer (140 mM NaCl, 4.7 mM KCl, 1.13 mM MgCl_2_, 1 mM CaCl_2_, 10 mM glucose, 10 mM HEPES, pH 7.2) containing 200 IU/mL collagenase (Worthington Biochemical Corp., Lakewood, NJ, USA) together with 0.5 mg/mL soybean trypsin inhibitor (Worthington Biochemical Corp.), and then incubated in a shaking water bath for 30 min at 37°C. The digested tissue was centrifuged through 50 mg/mL bovine serum albumin and washed twice with digestive buffer. Single cells or 5–8 cell clusters were resuspended in DMEM/F12 medium, counted using a hemocytometer and equally divided into groups as confirmed by protein estimations. Cells were allowed to recover at 37°C in a humidified 5% CO_2_ incubator, and cell viability was confirmed by trypan blue examination to be more than 95% before treatment. For treatment, cells were incubated with caerulein (10^−7^ M) together with or without lactose (0.5 mM) for 3 h before subsequent assays.

### Western Blot Analysis

Signaling proteins from acinar cell lysates were analyzed by Western blot analysis as previously described (25). Anti-bodies against phosphor-extracellular signal-regulated kinases (ERK)1/2 (#4370), total ERK (#4695), phosphor-p38 (#9211), total p38 (#9212), and p65 subunit of nuclear factor-κB (NF-κB) (#3033) were purchased from Cell Signaling Technology (Beverly, CA, USA).

### Statistical Analysis

Data are expressed as mean ± SEM. The parametric distribution of the results was confirmed using Kolmogorov–Smirnov test. Statistical analysis was performed by one-way or two-way (two independent variables) analysis of variance (ANOVA) followed by Tukey’s *post hoc* test using GraphPad Prism (version 5; GraphPad Software Inc., San Diego, CA, USA). *P* < 0.05 was considered to indicate a statistically significant difference.

## Results

### Lactose Treatment Alleviates the Severity of Caerulein-Induced AP

**Figure 1:**
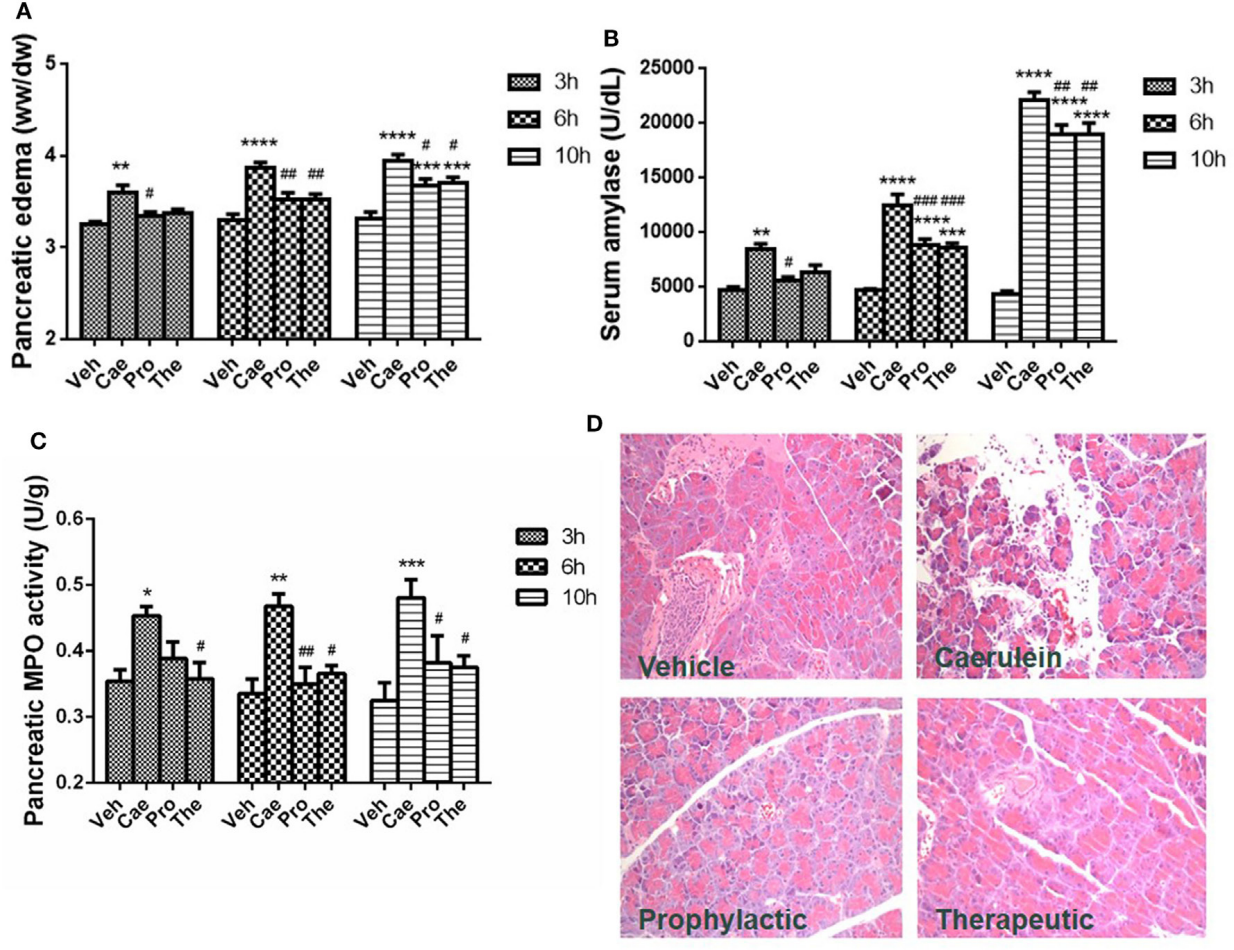
Prophylactic or therapeutic lactose treatment mitigates the severity of experimental acute pancreatitis in a time-related manner. Pancreatic edema **(A)** and serum amylase **(B)**, pancreatic myeloperoxidase activity **(C)**, and pancreatic histology **(D)** were determined. Vehicle (Veh): saline treatment. Caerulein (Cae): caerulein hyperstimulation treatment. Prophylactic: lactose administered 30 min before starting caerulein treatment. Therapeutic (The): lactose administered 1 h after starting caerulein treatment. Data are mean ± SEM from at least three independent experiments of six to eight independent mice in each group. *p < 0.05, **p < 0.01, ***p < 0.001, ****p < 0.0001 vs. corresponding Veh by two-way analysis of variance (ANOVA); ^#^*p* < 0.05, ^##^*p* < 0.01, ^###^*p* < 0.001 vs. corresponding Cae by two-way ANOVA.

Prophylactic or therapeutic administration of lactose (100 mg/kg) was evaluated in the most widely used mouse model of caerulein (50 µg/kg) hyperstimulation-induced AP. Mild, moderate, and severe AP were induced by 3, 6, and 10 hourly caerulein injections, respectively (8). Lactose treatment significantly reduced AP symptoms, including pancreatic edema, hyperamylasemia, and increased pancreatic MPO activities in a time-related manner. Either prophylactic or therapeutic lactose administration attenuated caerulein-induced pancreatic edema, increases in serum amylase and in pancreatic MPO levels (Figures 1A–C). Histological examination of pancreatic sections from mice with severe AP confirmed the protective effects by lactose treatment in AP against acinar cell injury, edema, and immune cell infiltration in the pancreas (Figure 1D). In addition, lactose administration protected AP-associated lung injury as evidenced by reduced lung MPO activities as compared to the caerulein group. Histological examination revealed decreased alveolar thickening and immune cell infiltration in the lungs of lactose-treated mice as compared to the AP group (Figures S1A,B in Supplementary Material). These findings suggest that lactose attenuates the severity of caerulein-induced AP and associated lung injury.

### Lactose Modulates Early Immune Cell Infiltration Into the Pancreas and Pancreatic Cytokine/Chemokine Production

**Figure 2:**
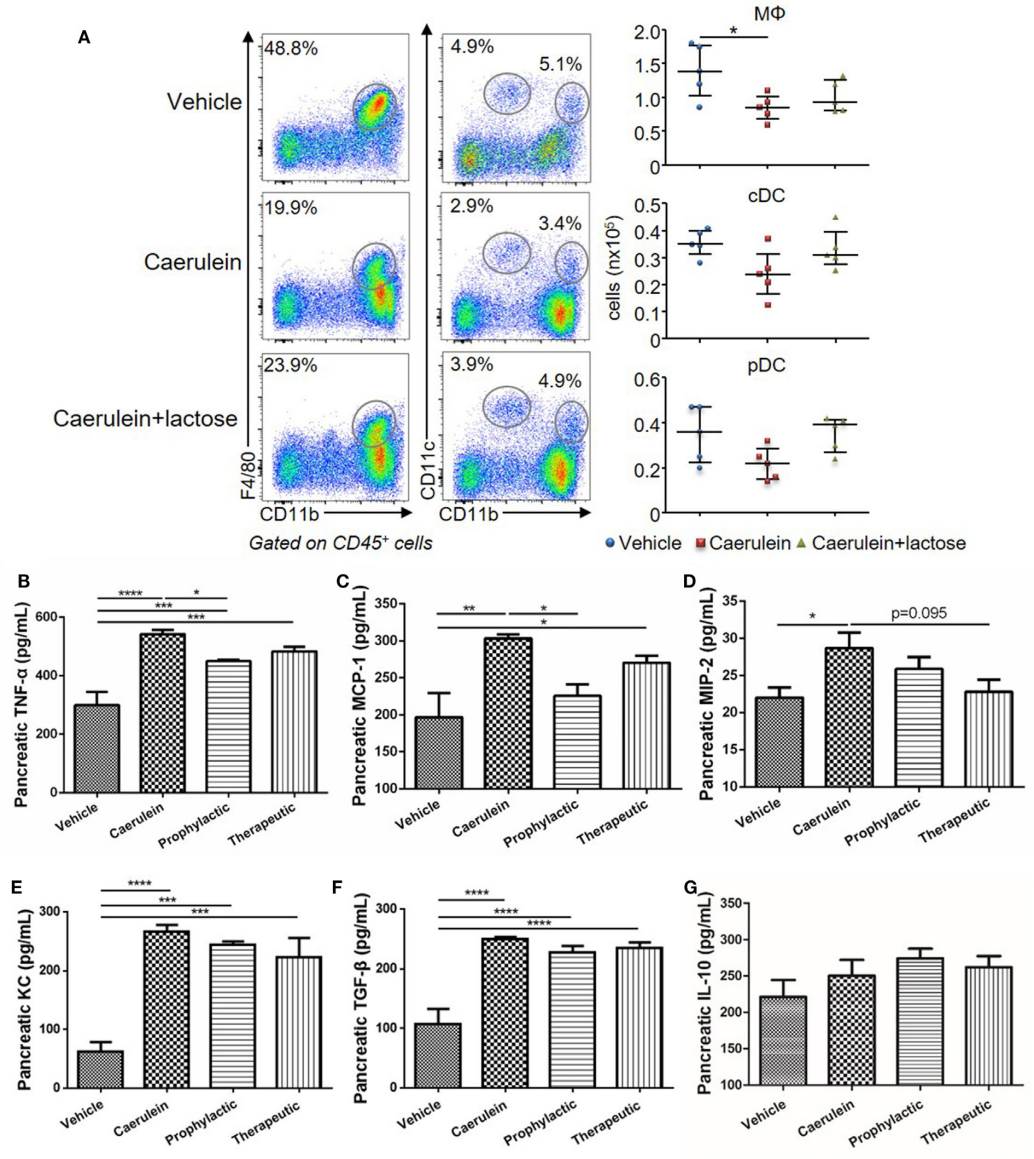
Lactose promotes early immune cell infiltration and a modulatory cytokine production profile in the pancreas. Female BALB/c mice were treated with caerulein together with or without lactose and 3 h later cells were recovered from the pancreas. **(A)** Frequency (gated on CD45^+^ cells) and absolute number of macrophages (CD11b^+^ F4/80^+^), conventional dendritic cells (F4/80^−^ CD11b^+^ CD11c^+^) and plasmacytoid dendritic cells (F4/80^−^ CD11b^+^ CD11c^med^) per pancreas were shown. Data are representative or median values ± interquartile range from four independent experiments with a minimum of five independent mice. **(B–G)** The levels of pancreatic tumor necrosis factor alpha, monocyte chemotactic protein-1, macrophage inflammatory protein-2, keratinocyte chemoattractant, transforming growth factor-β, and IL-10 production were shown. Data are mean ± SEM from at least three independent experiments of at least six independent mice in each group. *p < 0.05, **p < 0.01, ***p < 0.001 by one-way analysis of variance.

Dysregulated innate immune cell infiltration and a consequent imbalance between pro-inflammatory and anti-inflammatory cytokine/chemokine production are early hallmark pathological events of AP. To evaluate the effects of lactose on innate immune cell responses during early development of AP, BALB/c mice were treated with caerulein (50 µg/kg) together with or without lactose. After 3 h, the frequencies (gated on CD45^+^ cells) and absolute numbers of macrophages (CD11b^+^F4/80^+^), conventional dendritic cells (cDCs, F4/80^−^CD11b^+^CD11c^+^), and plasmacytoid DCs (pDCs, F4/80^−^CD11c^+^CD11b^med^) that infiltrated into the pancreas were analyzed. We found that the total number of macrophages was decreased in mice induced with AP, while the two subpopulations of DCs were not significantly altered as compared to untreated control mice (Figure 2A). Therefore, lactose treatment did not alter the total number of macrophages, pDCs, or cDCs (Figure 2A).

Inflammatory cytokines play an important role in the aggravation of AP and development of systemic inflammatory responses. We analyzed the modulatory effects of lactose on pancreatic cytokine production after 10 h induction of AP. Prophylactic or therapeutic treatment with lactose (100 mg/kg) resulted in decreased pancreatic MCP-1 production as compared to mice with severe AP, with more pronounced effects observed with prophylactic treatment (Figures 2B–D). In addition, lactose modulated the production of TNF-α and to a lesser extent macrophage inflammatory protein-2 production. In contrast, lactose did not alter AP-associated production of transforming growth factor-β (TGF-β), keratinocyte chemoattractant, or IL-10 (Figures 2E–G). Together, these data suggest that lactose treatment modulates the production of pro-inflammatory cytokines/chemokines in the pancreas.

### Lactose Modulates Neutrophil Infiltration and Activation During Early AP and Cytokine/Chemokine Production From Peripheral Neutrophils

**Figure 3:**
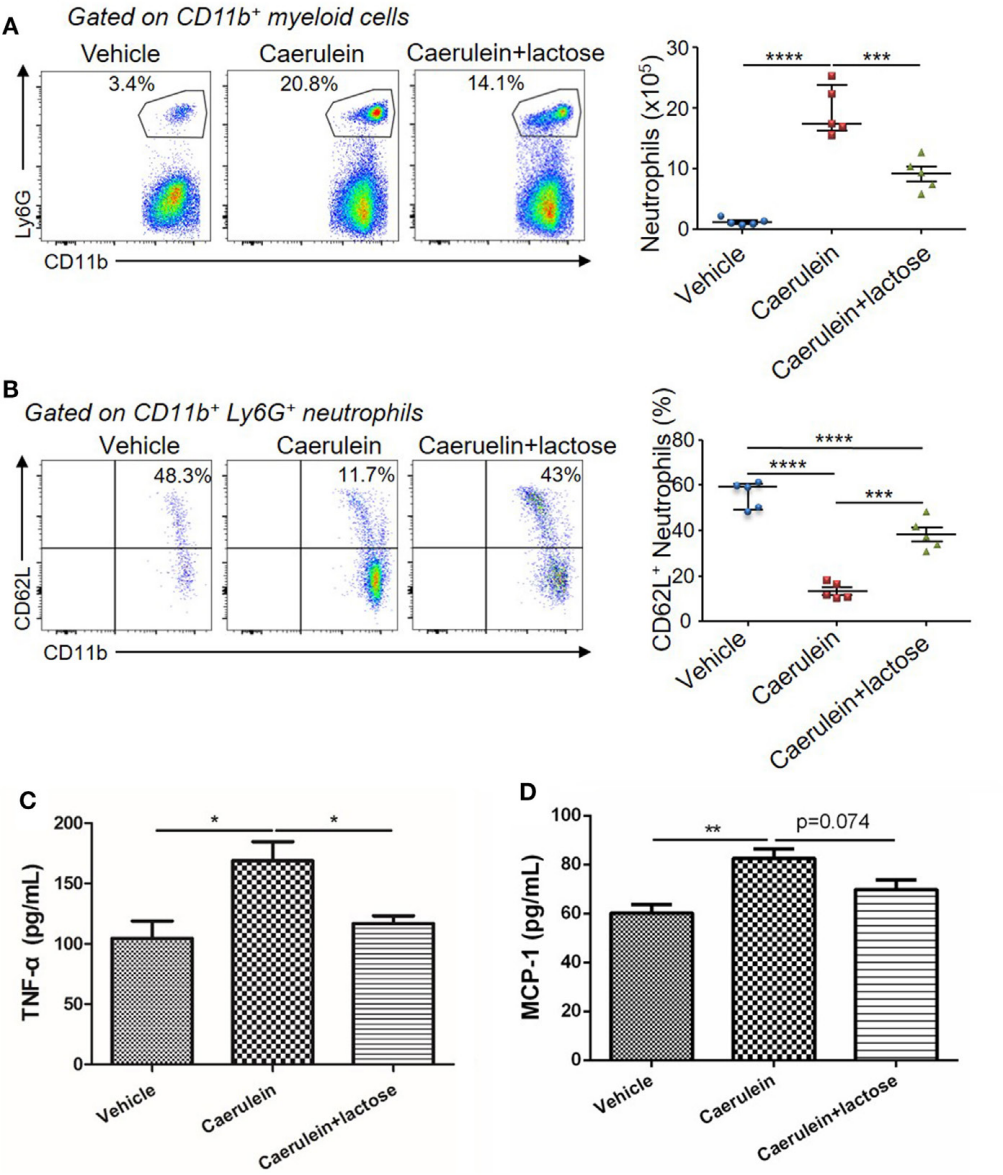
Lactose modulates neutrophil infiltration and activation during early acute pancreatitis and cytokine/chemokine production from peripheral neutrophils. Female BALB/c mice were treated with caerulein together with or without lactose and 3 h later cells were recovered from the pancreas. Cells were directly stained for the expression of CD45, Ly6G, CD11b, and CD62L. **(A)** The frequency and absolute number of neutrophils per pancreas were shown. **(B)** The frequency of CD62L^+^ neutrophils in the pancreas was shown. Results are representative or median values ± interquartile range from two independent experiments with a minimum of five independent mice. **(C,D)** Peripheral neutrophils were isolated, and incubated with lipopolysaccharides (1 µg/ml) for 3 h and the levels of tumor necrosis factor alpha **(C)** and monocyte chemotactic protein-1 **(D)** were analyzed by ELISA. Data are mean ± SEM from at least three independent experiments of at least five independent mice in each group. *p < 0.05, **p < 0.01 by one-way analysis of variance.

Further analysis of the infiltrated neutrophils (CD11b^+^Ly6G^+^) revealed that a large number of CD11b^+^Ly6G^+^ neutrophils were recruited to the pancreas 3 h after AP induction. Treatment with lactose resulted in attenuated infiltrating neutrophils (Figure 3A) and a higher CD62L^+^ proportion of infiltrated neutrophils (Figure 3B). We analyzed peripheral neutrophils in parallel and found that the levels of TNF-α production by peripheral neutrophils were significantly reduced in lactose-treated group as compared to the AP group (Figures 3C,D). Together, our results demonstrate that lactose modulates early neutrophil infiltration and activation in the pancreas, as well as functional cytokine production by peripheral neutrophils during AP.

### Lactose Modulates Macrophage Infiltration, Phenotype Conversion, and Cytokine Production During Early AP

**Figure 4:**
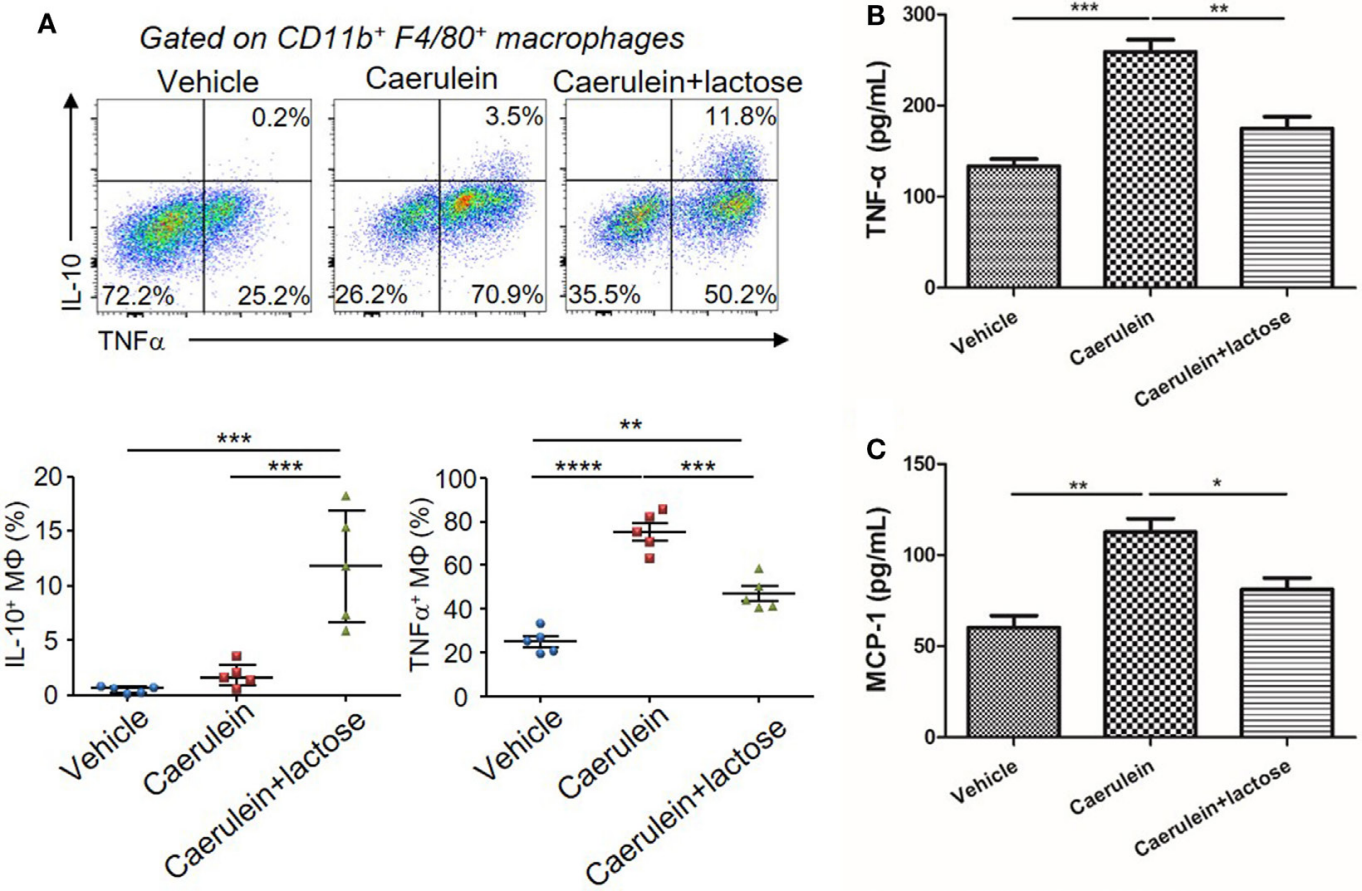
Lactose modulates macrophage infiltration, phenotypes and cytokine production during early acute pancreatitis. Female BALB/c mice were treated with caerulein together with or without lactose and 3 h later cells were recovered from the pancreas. **(A)** Pancreatic cells were stimulated 4 h with LPS (1 µg/ml) in the presence of brefeldin A and then stained for IL-10 and tumor necrosis factor alpha (TNF-α). Data are representative or median values ± interquartile range from two independent experiments with a minimum of five independent mice. **(B,C)** Peritoneal macrophages were isolated from peritoneal exudate cells followed by a recovery period and then stimulated with lipopolysaccharides (1 µg/ml) for 3 h and the levels of TNF-α **(B)** and monocyte chemotactic protein-1 **(C)** production were determined. Results are mean ± SEM from at least three independent experiments of at least five independent mice in each group. *p < 0.05, **p < 0.01, ***p < 0.001 by one-way analysis of variance.

In addition to neutrophils, macrophages represent another major source of inflammatory mediators in AP. Hence, we examined the infiltration and phenotypes of macrophages together with analyses of associated cytokine/chemokine production. AP induction was accompanied by an increase of TNF-α^+^ macrophages in the pancreas. Treatment with lactose resulted in increased IL-10^+^ macrophages and decreased TNF-α^+^ macrophages in the pancreas as compared to caerulein-induced AP mice (Figure 4A). In parallel, primary peritoneal macrophages were isolated and we found that lactose suppressed LPS induced elevation of TNF-α and MCP-1 in peritoneal macrophages (Figures 4B,C). These findings indicate that lactose exhibits regulatory effects on pancreatic macrophages, causing reduced pancreatic infiltration of these cells and inducing a phenotypic switch from an inflammatory toward a regulatory state.

In addition to innate immune cells, the effects of lactose on lymphocyte infiltration were also examined. Pancreatic B and T cells were not significantly changed upon AP induction, although a trend of decrease (*p* = 0.055) was observed for T cells. Lactose treatment did not affect T cell count but mildly reduced B cells (*p* = 0.039) (Figure S2 in Supplementary Material).

### Lactose Attenuates Caerulein-Induced MCP-1 Production and Activates ERK1/2-p65 Signaling Pathway in Pancreatic Acinar Cells

**Figure 5:**
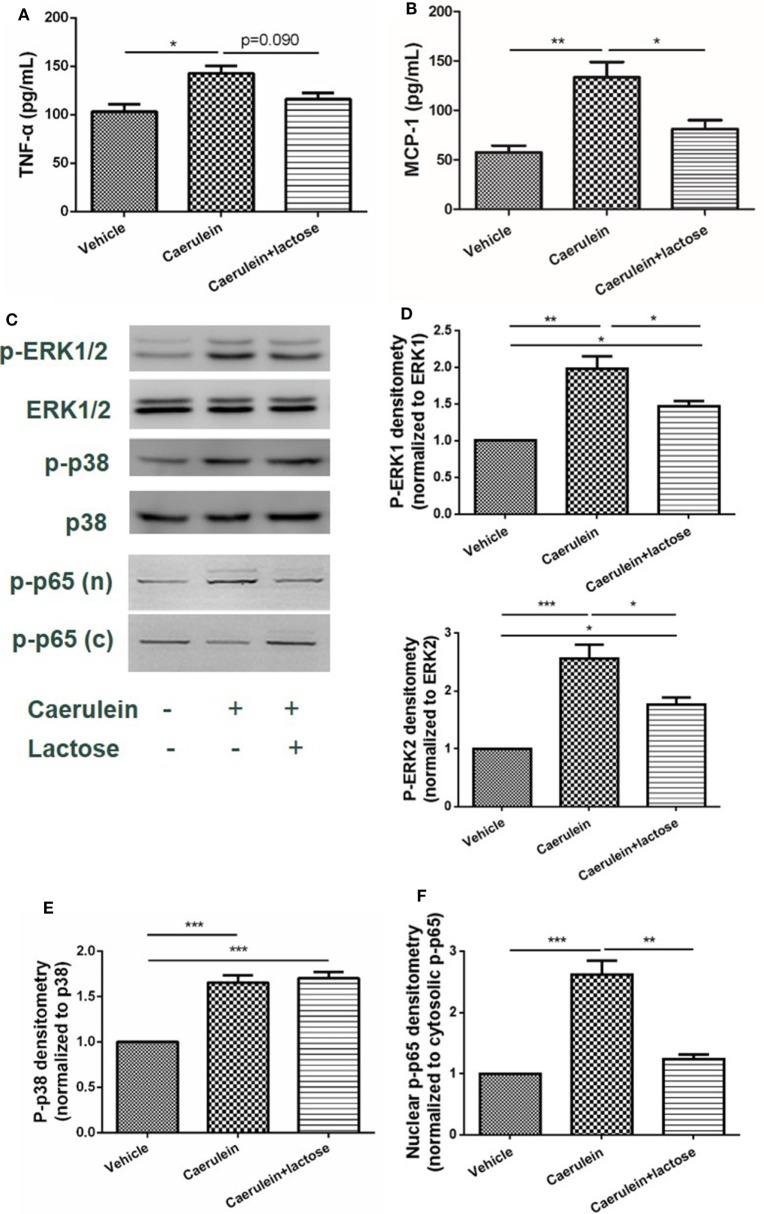
Lactose attenuates caerulein-induced cytokine/chemokine production and p-extracellular signal-regulated kinases(ERK)1/2-NF-κB p65 signaling pathway activation in pancreatic acinar cells. Acinar cells were isolated from fresh pancreas and treated with caerulein (10^−7^ M) together with or without 0.5 mM lactose. Shown are the levels of tumor necrosis factor alpha **(A)** and monocyte chemotactic protein-1 **(B)** production by ELISA and Western blot analysis **(C)** of p-ERK1/2, ERK1/2, p-p38, p38, nuclear (n), and cytosolic (c) p-NF-κB p65 and densitometry analysis **(D–F)**. Results are mean ± SEM from at least three independent experiments of at least three independent mice in each group. *p < 0.05, **p < 0.01 ***p < 0.001 by one-way analysis of variance.

Damaged pancreatic acinar cells produce inflammatory mediators that mediate local inflammation. Earlier studies have suggested that mitogen-activated protein (MAP) kinase and NF-κB signaling pathway participate in the pathophysiology of AP to induce the expression and release of inflammatory cytokines and chemokines (24). Using primary acinar preparations, we observed inhibitory effects of lactose (0.5 mM) on caerulein (10^−7^ M) induced MCP-1 production, but not TNF-α (Figures 5A,B). Furthermore, ERK1/2 was phosphorylated and activation in isolated acinar cells stimulated with caerulein, and lactose treatment downregulated p-ERK1/2 expression (Figures 5C,D). In contrast, either treatment with caerulein alone or with lactose did not alter p38 activation (Figures 5C,E). Further examination on the transcription factor NF-κB showed that lactose reversed caerulein-induced nuclear translocation and activation of NF-κB p65 subunit (Figures 5C,F). These results indicate that lactose suppresses ERK1/2 and NF-κB p65 signaling, leading to inhibition of cytokine production in pancreatic acinar cells.

### Galectin-3 Antagonism Mimicking Lactose Modulates Neutrophil Recruitment and Activation in the Pancreas After AP Induction

**Figure 6:**
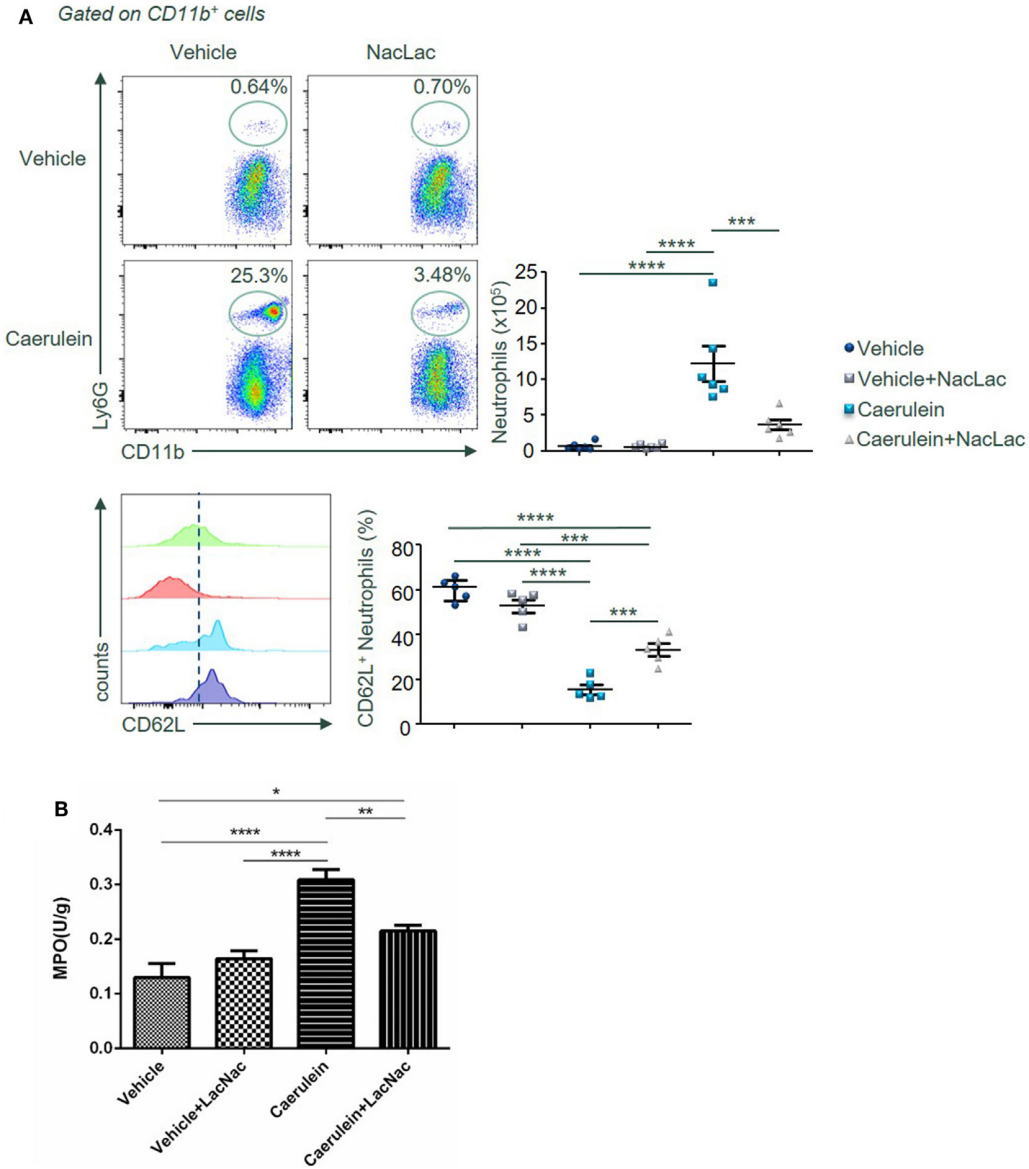
An antagonist of galectin-3 mimicking lactose suppresses neutrophil recruitment and activation in the pancreas after AP induction. Female BALB/c mice were treated with caerulein together with or without *N*-acetyl-d-lactosamine and 3 h later cells were recovered from the pancreas. **(A)** The frequency of CD62L^+^ neutrophils in the pancreas was shown. Results are representative or median values ± interquartile range from three independent experiments with a minimum of five to six independent mice. **(B)** Pancreatic myeloperoxidase activities were shown. Results are mean ± SEM from two independent experiments of at least five independent mice in each group. *p < 0.01, **p < 0.01, ***p < 0.001, ****p < 0.0001 by one-way analysis of variance.

Last, we examined potential molecular targets of lactose for its immunomodulatory effects during AP. Earlier, lactose has been found to inhibit galectin-3 binding and galectin-3 is known to promote activation of inflammatory macrophages and neutrophil migration (26–28). BALB/c female mice were treated with caerulein (50 µg/kg) alone or together with the galectin-3 inhibitor *N*-acetyl-d-lactosamine. Neutrophil infiltration and activation as well as the neutrophil-derived enzyme MPO activity were subsequently examined. Our results demonstrate that *N*-acetyl-d-lactosamine mimicking the effects of lactose, significantly decreased the frequency of CD11b^+^Ly6G^+^ neutrophils (Figure 6A). Accordingly, we found that MPO activity was reduced in *N*-acetyl-d-lactosamine-treated group as compared to caerulein only treated group (Figure 6B). Together, these data suggest that galectin-3 may be the potential downstream molecular target of lactose.

## Discussion

**Figure 7:**
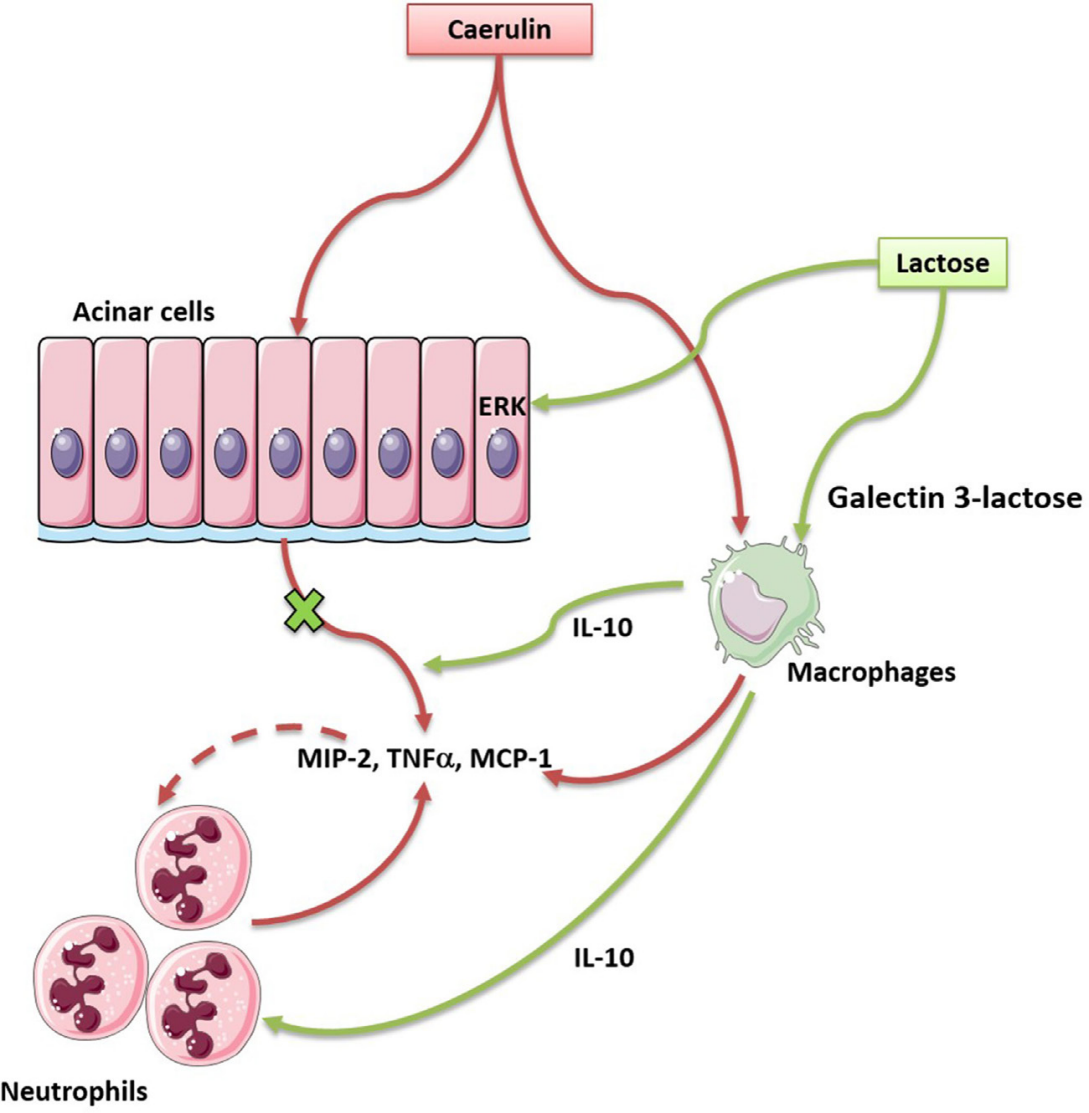
Lactose promotes modulatory immune responses during caerulein-induced experimental acute pancreatitis (AP) by antagonizing galectin-3. In our study, lactose protects mice against AP induced by caerulein *via* regulating the infiltration of neutrophils and macrophages, reducing the release of inflammatory mediators especially the cytokine tumor necrosis factor alpha and the chemokine monocyte chemotactic protein-1, inhibiting ERK1/2–p65 activation. In addition, lactose treatment promotes IL-10-producing macrophages in the pancreas and may bind galectin-3 to alleviate the severity of AP.

The current study demonstrates that lactose exerts immunomodulatory effects to alleviate experimental AP. The proposed mechanisms of action are: lactose reverses AP-induced infiltration of activated neutrophils and increases regulatory (IL-10-producing) vs. inflammatory (TNF-α-producing) macrophage ratio. Lactose directly attenuates caerulein-induced inflammatory signaling pathway and MCP-1 production in acinar cells. This study provides evidence that lactose treatment limits local inflammation and prevents exacerbated inflammatory responses during AP (Figure 7).

Lactose has been documented to possess immune modulatory effects. Lactose, isolated from human breast milk, has been found to induce the cathelicidin antimicrobial peptide (CAMP) gene encoding the only human *CAMP* LL-37 in THP-1 monocytes, macrophages, as well as in the colonic epithelial cells HT-29 and T84 (16), suggesting its importance in neonatal innate immunity and intestinal homeostasis. Alpha (α)-lactose-treated mice exhibited enhanced acute and memory responses against mucosal pathogens (29), indicating lactose treatment as a convenient and inexpensive approach in mucosal immunopathology. In addition, milk oligosaccharide derivative sialyl(α 2,3) lactose has been shown to promote intestinal inflammation through interaction with toll-like receptor 4 in a mouse model of colitis (30). In this study, supplementation with 25 mM lactose did not result in inflammation in mice. Oligosaccharides may, therefore, play a role in the regulation of mucosal immunity. In line with these, lactose has been suggested as a potential alternative to growth-promoting antibiotic in controlling necrotic enteritis (19). Recently, α-lactose has been shown to improve the survival of mice with sepsis by tim-3 blockade to prevent natural killer T (NKT) cell apoptosis (17), supporting its role in NKT cell modulation and systemic immunity. On the other hand, lactose has been found to inhibit human CD4^+^CD25^+^CD127^−^ regulatory T (T_reg_)-mediated suppression of Th1 and Th17 immune responses *in vitro* (15). These results may be associated with the fact that oligosaccharides reach lamina propria and that lactose can induce harmful inflammatory responses by disrupting T_reg_-mediated regulation in individuals susceptible to chronic inflammatory diseases. Another recent study has demonstrated that 300 mM of α-lactose injected into mice infected with *Plasmodium berghei* exacerbate pulmonary immunopathology by blocking the interaction of galectin-9 and its receptor (18). It can be postulated that the anti- or pro-inflammatory effects of lactose may be related to its concentrations and primary molecular targets in the disease context. For example, pro-inflammatory effects of lactose have been suggested *via* galectin-9 antagonism to abrogate downstream tim-3-mediated immune regulation (15, 31). In this study, we found that lactose and the galectin-3 inhibitor *N*-acetyl-d-lactosamine significantly reduced neutrophils infiltration during caerulein induced development of AP, suggesting that lactose may act *via* antagonizing galectin-3. The concentration of lactose (100 mg/kg) was selected from earlier *in vivo* studies to be relevant to its nutritional values (30, 32, 33), and also determined by preliminary experiments as the lowest effective concentration. The route of administration, i.p., ensures that the observed effects are those of lactose and not its metabolites or digested products (e.g., by avoiding metabolism by the liver) and is relevant to the parenteral route in clinical nutrition (34). Our data are in line with previous studies supporting that lactose may bind to and antagonize galectin-3 (35). Galectin-3 has been shown to mediate neutrophil migration (27) and to interact with naive and primed neutrophils, promoting neutrophil-mediated innate immune responses (36). Furthermore, galectin-3 also attracts monocytes and macrophages (20), and induces production of inflammatory mediators in various conditions (21, 28, 37, 38). Ours together with earlier studies provide evidence that milk oligosaccharides acting *via* its intracellular molecular target play an important role in the regulation of mucosal immunity in the pancreas.

Conventional views and recent studies have highlighted innate immunity and acinar cell inflammation in the pathogenesis of AP (7). Immune cells and derived inflammatory mediators, importantly cytokines/chemokines determine the disease severity (6). We observed that caerulein hyperstimulation caused activation and infiltration of neutrophils and inflammatory macrophages, which may be reversed by lactose treatment. AP induction was associated with an increase in CD11b^+^Ly6G^+^ neutrophils and a decrease in the frequency (gated on CD11b^+^Ly6G^+^) of CD62L^+^ neutrophils. CD62L is an adhesion molecule present on granulocytes, monocytes, and lymphocytes and its regulation is closely related with the disease states (9, 39). We have found that caerulein-induced AP is associated with CD62L shedding from the cell surface of activated neutrophils and lactose treatment restored its expression. In view of the role of CD62L in leukocyte rolling along endothelial cells during migration, its upregulation by lactose may be to protect against neutrophil trafficking, neutrophil-mediated pro-inflammatory responses, and manifestation of systemic inflammatory responses. Similar observations have been reported in acute respiratory distress syndrome, where blood and alveolar neutrophils of patients exhibited increased CD11b and reduced CD62L expression compared to healthy circulating cells (9). Reversion of this phenotypic change by lactose indicates that its modulatory effect is associated with decreased infiltration of activated neutrophils. Furthermore, we have found that caerulein induction of AP results in increased frequency of TNF-α-producing macrophages, and lactose treatment caused decreased frequency of TNF-α^+^ macrophages and increased frequency of IL-10^+^ macrophages. Increased pancreatic Ly6C^hi^ monocytes/macrophages are shown to be positively related to the severity of AP, which is dependent upon the expression of TNF-α by these cells (10). Interfering with M2 polarization has been reported to enhance the inflammatory status of macrophages during pancreatitis (11), and IL-10 production has been shown to impair neutrophil recruitment in inflammatory and infectious conditions (40, 41). Along with reduced infiltration of activated neutrophils and macrophages in lactose-treated mice, MCP-1 and TNF-α levels were found to be suppressed in pancreatic tissues, in addition to peripheral neutrophils and peritoneal macrophages. Selected cytokine/chemokine modulation by lactose may result from an overall effect on differential regulation of their cellular sources in the pancreas. The resultant reduction of cytokine and chemokine production prevents augmented inflammatory responses from a positive inflammatory feedback circuit (42).

Acinar cell inflammation with activation of MAP kinases and the transcription factor NF-κB is a key pathological event in early AP (2, 24, 25, 43). Supraphysiological concentrations of caerulein induces ERK1/2 phosphorylation and activation in pancreatic acini (44). NF-κB transactivation aggravates acute pancreatic inflammatory responses and MAP kinases regulate the activation of NF-κB by mediating phosphorylation of its inhibitory-κB (IκB) protein, to allow NF-κB translocation into the nucleus followed by inflammatory gene expression (44–46). Here, we found that MAP kinases ERK1/2, but not p38 activation was inhibited by lactose in acinar cells, concomitant with an attenuated NF-κB p65 nuclear translocation and activation. It can be speculated that different upstream MAP kinases are regulated by lactose. Consequently, caerulein-induced production of TNF-α and MCP-1 was reduced by lactose, which is in agreement with previous studies, showing that inhibition of MAP kinase signaling in acinar cells leads to downregulation of chemokines including MCP-1 (24). Our data fully imply that lactose regulates the development of AP, which is dependent on the activation of ERK1/2-NF-κB p65 signaling pathway and the release of inflammatory mediators.

In summary, our results show that lactose has positive effects on innate immune cell and acinar cell responses against caerulein-induced AP, revealing the interplay between this nutrient with pancreatic immunity and suggests the potential molecular target during this condition. As a curative treatment for AP remains obscure, the current study provides basic experimental evidence to develop nutritional intervention strategies to improve clinical management of AP. The milk oligosaccharide lactose with anti-inflammatory properties during AP may be considered as a convenient, safe (for tolerant individuals), and value-added nutrient to control local inflammation and lower the risk of AP-associated systemic complications.

## Ethics Statement

All animal-related experimental protocols were approved by the Institutional Animal Ethics Committee of Jiangnan University in compliance with the recommendations of national and international guidelines for the Care and Use of Laboratory Animals, and were performed in accordance with the guidelines therein. Five to eight animals were used in each group for the experiments and have been specifically indicated in the respective figure legends.

## Author Contributions

JS, JD, and LLP conceived the study project and designed experiments. LLP, YYD, and JD performed experiments and analyzed the data, with general assistance from CW, JL, WN, and QY on animal models, Western blot, and statistical analysis. RW, MB, BA, and GHG provided intellectual inputs and contributed to data acquisition. LLP, JS, and YYD drafted the manuscript. All authors contributed to the interpretation of the experiments and critically reviewed the manuscript. All authors gave final approval of the work.

## Conflict of Interest Statement

The authors declare that the research was conducted in the absence of any commercial or financial relationships that could be construed as a potential conflict of interest.

## Supplementary Material

Figure S1 Lactose treatment modulates the severity of experimental AP-associated lung injury. Lung myeloperoxidase activity **(A)** and histology **(B)** were determined. Vehicle: saline treatment. Caerulein: caerulein hyperstimulation treatment. Prophylactic: lactose administered 30 min before starting caerulein treatment. Therapeutic: lactose administered 1 h after starting caerulein treatment. Data are mean ± SEM from at least three independent experiments of six to eight independent mice in each group. ***p* < 0.01, ****p* < 0.001 vs. vehicle by two-way analysis of variance.

Figure S2 Effects of lactose on pancreatic B and T cells following acute pancreatitis induction. Female BALB/c mice were treated with caerulein together with or without lactose and 3 h later cells were recovered from the pancreas. Cells were directly stained for the expression of CD45, Ly6G, CD11b, CD19, and TCRβ. B and T cell numbers per pancreas were shown. Results are representative or median values ± interquartile range from two independent experiments with a minimum of five independent mice.

## Abbreviations

AP, acute pancreatitis; *CAMP*, cathelicidin antimicrobial peptide; cDC, conventional dendritic cells; ERK, extracellular signal-regulated kinases; H&E, hematoxylin and eosin; IL-10, interleukin-10; KC, keratinocyte chemoattractant; LPS, lipopolysaccharides; MAP, mitogen-activated protein; MCP-1, monocyte chemotactic protein-1; MIP-2, macrophage inflammatory protein-2; MPO, myeloperoxidase; NF-κB, nuclear factor-kappaB; NKT, natural killer T cells; pDC, plasmacytoid dendritic cells; T_reg_, regulatory T cells; TGF-β, transforming growth factor-β; TNF-α, tumor necrosis factor alpha.
