# Supplementary figures and images for: Lactose Induces Phenotypic and Functional Changes of Neutrophils and Macrophages to Alleviate Acute Pancreatitis in Mice
Source: Front Immunol. 2018 Apr 17;9:751. doi: 10.3389/fimmu.2018.00751 (PMC5913286; doi:10.3389/fimmu.2018.00751)

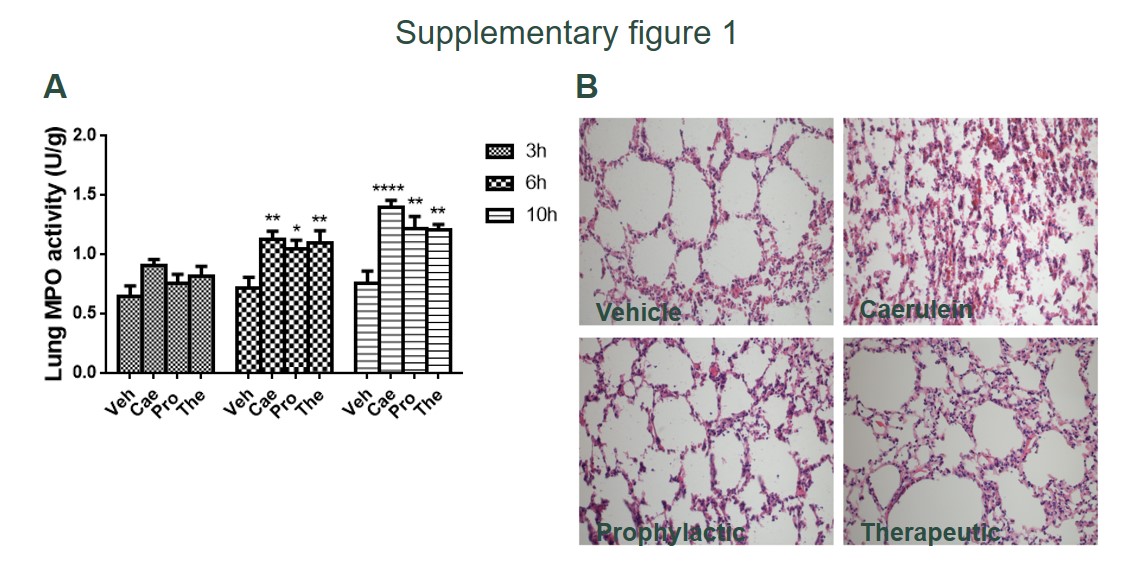

Supplement: Figure S1 — Lactose treatment modulates the severity of experimental AP-associated lung injury. Lung myeloperoxidase activity (A) and histology (B) were determined. Vehicle: saline treatment. Caerulein: caerulein hyperstimulation treatment. Prophylactic: lactose administered 30 min before starting caerulein treatment. Therapeutic: lactose administered 1 h after starting caerulein treatment. Data are mean ± SEM from at least three independent experiments of six to eight independent mice in each group. **p < 0.01, ***p < 0.001 vs. vehicle by two-way analysis of variance. [file image_1.jpeg]

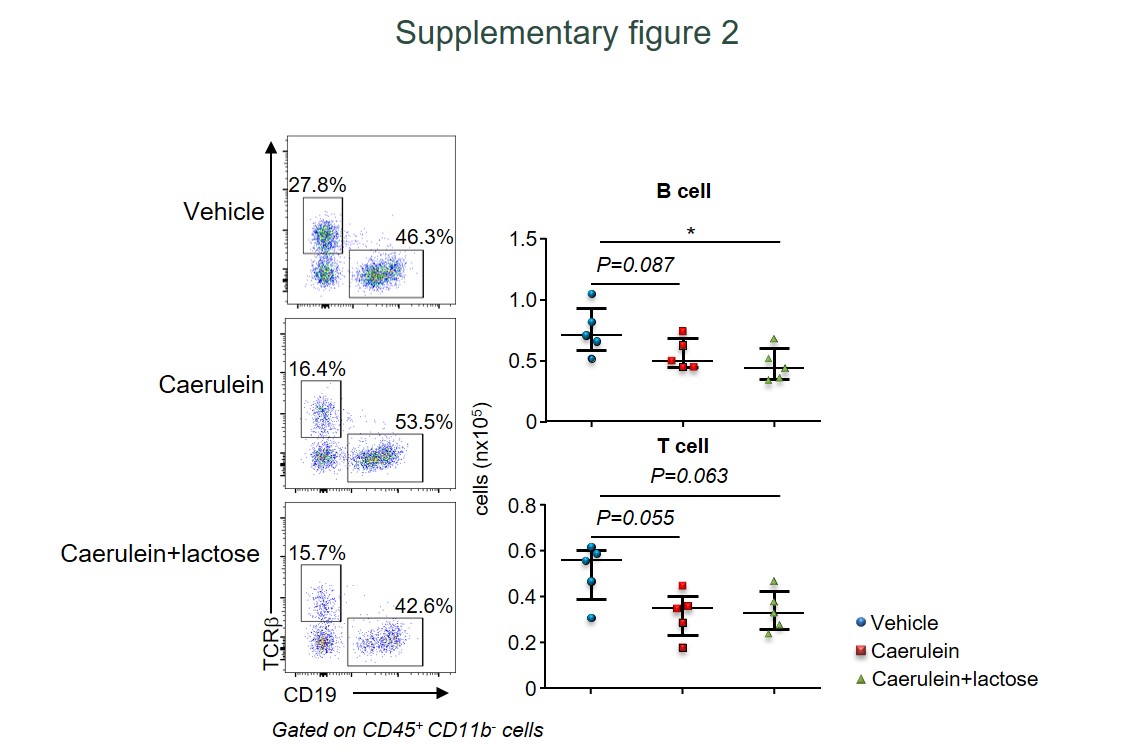

Supplement: Figure S2 — Effects of lactose on pancreatic B and T cells following acute pancreatitis induction. Female BALB/c mice were treated with caerulein together with or without lactose and 3 h later cells were recovered from the pancreas. Cells were directly stained for the expression of CD45, Ly6G, CD11b, CD19, and TCRβ. B and T cell numbers per pancreas were shown. Results are representative or median values ± interquartile range from two independent experiments with a minimum of five independent mice. [file image_2.jpeg]
